# 1664. Impact of a Midline Catheter Prioritization Project on Central Venous Access and Central Line Associated Bloodstream Infections at an Urban Safety-net Community Hospital

**DOI:** 10.1093/ofid/ofac492.130

**Published:** 2022-12-15

**Authors:** Alfredo J Mena Lora, Brenna Lindsey, Stephanie Echeverria, Mirza Ali, Candice Krill, Eden Takhsh, Susan C Bleasdale

**Affiliations:** University of Illinois Chicago, Saint Anthony Hospital, CHICAGO, Illinois; University of Illinois at Chicago, Chicago, Illinois; Saint Anthony Hospital, Chicago, Illinois; Saint Anthony Hospital, Chicago, Illinois; Saint Anthony Hospital, Chicago, Illinois; Saint Anthony Hospital, Chicago, Illinois; University of Illinois at Chicago, Chicago, Illinois

## Abstract

**Background:**

The use of peripherally inserted central catheters (PICCs) has increased in the past decade. PICCs are central lines (CLs) commonly used for venous access. Midline catheters (MLs) can provide access when the need for a CL, such as vasopressors, is no longer present. MLs have a lower rate of BSI compared to PICCs and CLs, while providing dwell times comparable to PICCs. We established a project prioritizing ML use.

**Methods:**

This is a quasi-experimental study in a 151-bed safety net community hospital. The pre-intervention period was January-December 2018 and post period was January 2019-December 2021. MLs were prioritized when new PICCs are requested without CL indications, such as total parenteral nutrition, hyperosmolar solutions and vasopressors. PICCs and CLs are transitioned to a ML once indications are no longer met and peripheral IVs are not feasible. Data on utilization and complications, such as deep venous thrombus (DVT) and BSIs, were reviewed and compared.

**Results:**

A total of 63 peripherally inserted lines occurred prior to the intervention, of which 55 (87%) were PICC and 8 (13%) were ML (Figure 1). Post-intervention, 76 lines were placed the first year, of which 48 were ML (63%). This upward trend was sustained throughout the pandemic, with 116 lines in 2020 (80% ML) and 96 in 2021 (88% ML). No BSIs occurred during the pre-intervention and first post-intervention year. During the pandemic, 8 BSIs in MLs and 3 in PICCs occurred. The most common organism was Candida (Figure 2). The majority had COVID-19 (72%) and all (100%) BSIs were in the setting of shock. Case review demonstrated suspected secondary sources other than central venous catheters (CVCs). All BSIs with ML would have met NHSN criteria if CL present. No upper extremity DVTs were found.

**Conclusion:**

A midline prioritization project was successfully implemented and sustained during the COVID-19 pandemic. The decline of PICC use from 87% to 12% suggests use for access without CL needs. High acuity during the pandemic led to BSIs that were likely secondary to shock and complications of COVID-19. All cases would have met NHSN criteria for CLABSI. The cost of a CLABSI is estimated at $48,108. Thus, this midline prioritization project may have led to CLABSI avoidance and an estimated cost savings of $384,864.

**Disclosures:**

**All Authors**: No reported disclosures.

**Figure 1.**
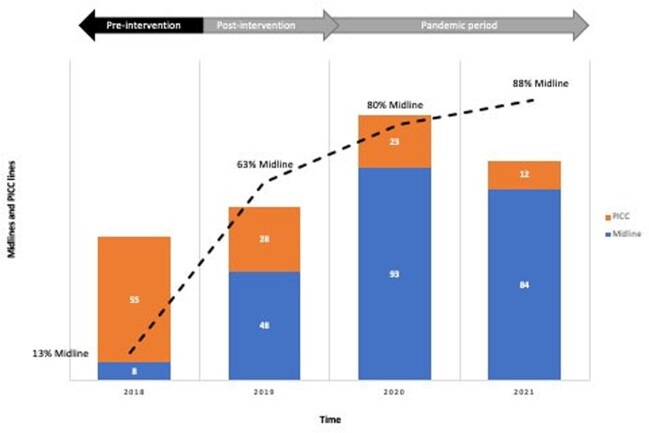
Midlines and PICC lines during our pre-intervention and post-intervention period.

**Figure 2.**
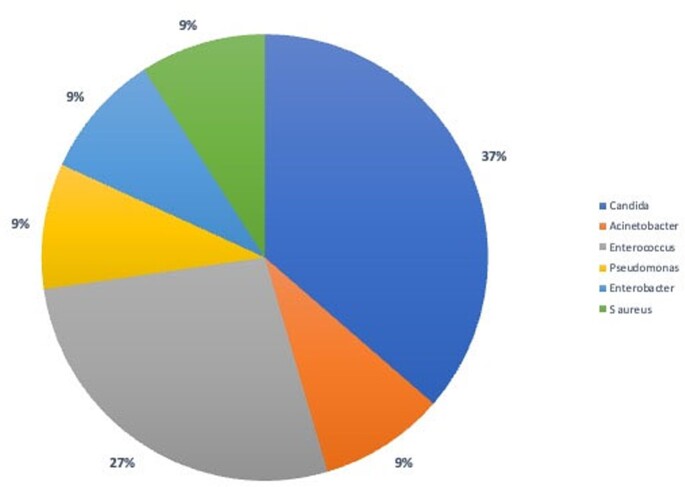
Organisms impicated in bloodstream infections associated with Midlines and PICC lines.

